# Neurological complications of *Mycoplasma pneumoniae* infection

**DOI:** 10.1590/0037-8682-0342-2025

**Published:** 2026-02-09

**Authors:** Conceição Pereira, Felipe Scortegagna, Diogo Goulart Corrêa, Felipe Pacheco

**Affiliations:** 1 Hospital Infantil Sabará, Departamento de Neurologia, São Paulo, SP, Brasil.; 2 Diagnósticos da América SA, Divisão de Neurorradiologia, São Paulo, SP, Brasil.; 3 Universidade do Estado do Rio de Janeiro, Departamento de Diagnóstico por Imagem, Disciplina de Radiologia, Rio de Janeiro, RJ, Brasil.; 4 Clínica de Diagnóstico por Imagem (CDPI)/DASA, Departamento de Radiologia, Rio de Janeiro, RJ, Brasil.; 5 Irmandade da Santa Casa de Misericórdia de São Paulo, Divisão de Neurorradiologia, São Paulo, SP, Brasil.

*Mycoplasma pneumoniae* is a common cause of respiratory tract infections^1^, and may also lead to various extrapulmonary manifestations, including neurological complications^2^. Herein, we present three cases of neurological involvement associated with *M. pneumoniae* infection. 

**FIGURE 1:**
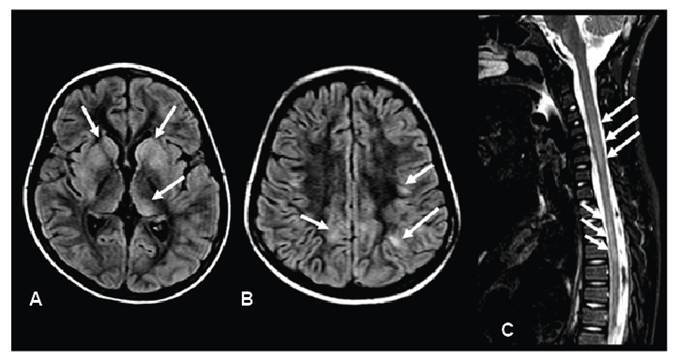
Brain magnetic resonance imaging (MRI) reveals multiple bilateral and asymmetrical lesions with hyperintense signal on FLAIR, involving the basal ganglia (**arrows in A**) and subcortical white matter (**arrows in B**), consistent with acute disseminated encephalomyelitis. Spine MRI demonstrates longitudinally extensive myelitis with hyperintense signal on T2-weighted imaging (**arrows in C**).

Case 1. A 7-year-old girl presented with seizures and reduced consciousness 3 weeks after completing amoxicillin treatment for tonsillitis. Brain and spinal magnetic resonance imaging (MRI) revealed lesions consistent with acute disseminated encephalomyelitis (Figure 1). Serum testing was positive for anti-myelin oligodendrocyte glycoprotein antibodies and for both immunoglobulin M (IgM) and IgG against *M. pneumoniae*. Cerebrospinal fluid (CSF) analysis demonstrated mild pleocytosis (77 lymphocytes/mm^3^) with normal protein and glucose levels. The patient was treated with immunoglobulin and methylprednisolone for 5 days and intravenous clarithromycin for 14 days, resulting in full recovery.

**FIGURE 2:**
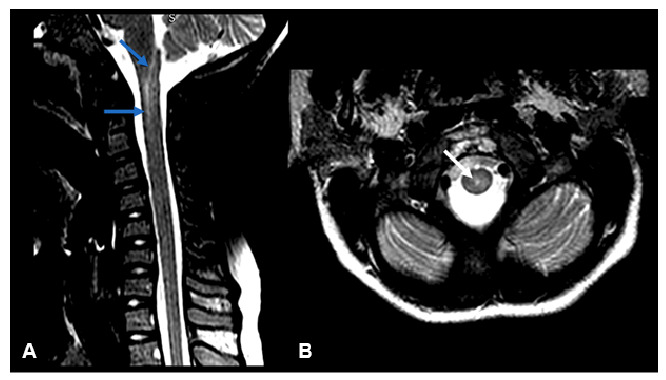
Spine magnetic resonance imaging (MRI) shows a hyperintense signal within the central spinal cord extending from the obex to the C2 level on T2-weighted imaging, visible in both sagittal (**arrows in A**) and axial (**arrow in B**) planes, consistent with myelitis. No gadolinium enhancement was observed. Brain MRI findings were normal.

Case 2. A 10-year-old boy presented with a 3-day history of lower limb paresis and neck stiffness, without preceding respiratory symptoms. Spinal MRI revealed myelitis extending from the obex to C2 (Figure 2). Serum testing was positive for *M. pneumoniae* IgM and IgG. CSF analysis showed pleocytosis (206 lymphocytes/mm^3^, 12 monocytes/mm^3^, and 7 neutrophils/mm^3^), elevated protein (110 mg/dl), and normal glucose. Treatment with clarithromycin for 14 days and methylprednisolone for 5 days, followed by prednisolone for 6 months, led to complete recovery.

**FIGURE 3:**
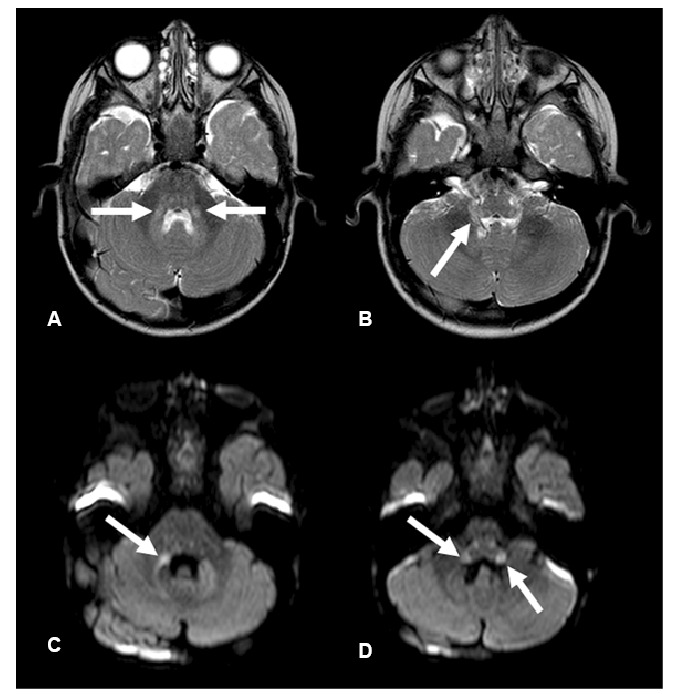
Brain magnetic resonance imaging demonstrates hyperintense lesions on T2-weighted imaging (**A and B**), with restricted diffusion (**C and D**), involving the pons and middle cerebellar peduncles (**arrows in A and C**), as well as the medulla and inferior cerebellar peduncles (**arrows in B and D**), consistent with rhombencephalitis.

Case 3. A 19-month-old boy presented with a 2-day history of fever and ataxic gait. Brain MRI revealed lesions consistent with rhombencephalitis (Figure 3). Serum testing was positive for *M. pneumoniae* IgM. The patient was treated with levofloxacin for 10 days, with complete resolution of symptoms.

The pathophysiology of *M. pneumoniae*-associated neurological complications remains incompletely understood. the following three mechanisms have been proposed, which may occur even in the absence of respiratory symptoms: (1) direct invasion, with bacterial presence at the site of inflammation (typically within 7 days of respiratory symptoms onset); (2) indirect immune-mediated processes involving autoimmunity or immune complexes (after 8 days of respiratory symptom onset, if present); and (3) vascular occlusion^2^ ^,^ ^3^. Encephalitis and myelitis may result from either direct invasion or immune-mediated mechanisms, whereas stroke is more commonly associated with vascular occlusion^4^.
